# Ankylosing spondylitis patients display altered dendritic cell and T cell populations that implicate pathogenic roles for the IL-23 cytokine axis and intestinal inflammation

**DOI:** 10.1093/rheumatology/kev245

**Published:** 2015-08-28

**Authors:** Pamela B. Wright, Anne McEntegart, David McCarey, Iain B. McInnes, Stefan Siebert, Simon W. F. Milling

**Affiliations:** ^1^Institute of Infection, Immunity and Inflammation, College of Medical, Veterinary and Life Sciences, University of Glasgow and; ^2^Department of Rheumatology, Queen Elizabeth Building, Glasgow Royal Infirmary, Glasgow, UK

**Keywords:** dendritic cells, CD4^+^ T cells, ankylosing spondylitis, inflammation, CD14^−^ CD16^+^ mononuclear cells

## Abstract

**Objective.** AS is a systemic inflammatory disease of the SpA family. Polymorphisms at loci including HLA-B27, IL-23R and ERAP-1 directly implicate immune mechanisms in AS pathogenesis. Previously, in an SpA model, we identified HLA-B27–mediated effects on dendritic cells that promoted disease-associated Th17 cells. Here we extend these studies to AS patients using deep immunophenotyping of candidate pathogenic cell populations. The aim of our study was to functionally characterize the immune populations mediating AS pathology.

**Methods.** Using 11-parameter flow cytometry, we characterized the phenotype and functions of lymphocyte and myeloid cells from peripheral blood, and the synovial phenotype of AS patients and age-matched healthy controls.

**Results.** Significantly fewer circulating CD1c-expressing dendritic cells were observed in AS patients, offset by an increase in CD14^−^ CD16^+^ mononuclear cells. *Ex vivo* functional analysis revealed that this latter population induced CCR6 expression and promoted secretion of IL-1β and IL-6 when co-cultured with naive CD4^+^ T cells. Additionally, systemic inflammation in AS patients significantly correlated with increased proportions of activated CCR9^+^ CD4^+^ T cells.

**Conclusion.** CD14^−^ CD16^+^ mononuclear cells may contribute to AS by promoting Th17 responses, and antigen-presenting cells of mucosal origin are likely to contribute to systemic inflammation in AS.

Rheumatology key messages

- The AS immunological signature consists of altered myeloid (CD1c^+^ : CD16^+^) and lymphocyte (CCR6 : CXCR3) profiles.
- CD14^−^ CD16^+^ mononuclear cells may support Th17-mediated AS disease pathology through CCR6, IL-1β and IL-6 induction.
- The intestinal environment may drive systemic inflammation in AS patients in a CCR9-dependent manner.

## Introduction

AS is a chronic arthropathy of the axial and peripheral skeleton, characterized by inflammation and abnormal bone and entheseal remodelling. AS is recognized as the prototypic disease of a larger SpA group of conditions that share genetic, pathophysiological and clinical features. Although transformed by the introduction of TNF inhibitors a decade ago, current therapeutics remain inadequate for a substantial proportion of patients, offering a compelling case for improved pathophysiological discovery. Large genome-wide association studies have recently contributed several novel loci and hence novel candidate pathways in this regard. There is particular interest in the confirmed associations with IL-23R, ERAP1 and STAT3 [1–3]. Taken together with the provisional clinical benefit accrued upon inhibition of the IL-17A pathway in clinical trials in AS patients [4], this suggests that novel immune-mediated pathways beyond TNF may now be formally implicated in pathogenesis.

Prior mechanistic studies have relied heavily on data generated using animal models. Experiments using transgenic rats overexpressing the human HLA-B27 and β2-microglobulin genes (B27-TG) have, for instance, implicated CD4^+^ T cells [5, 6], myeloid cells [7, 8] and endoplasmic reticulum (ER) stress [9] in the development of SpA. There are as yet few data informing analogous mechanisms operating in patients. Given that HLA-B27 is an MHC class I molecule, it is intriguing that disease in B27-TG rats occurs independently of CD8^+^ T cells [6]. Moreover CD4^+^ T cells expanded in AS patient peripheral blood are enriched for a population of Th17 phenotype [10–12]. Dendritic cells (DCs), which regulate T cell responses, have been strongly implicated in SpA pathogenesis in B27-TG rats [7, 8, 13]. Parallel studies in patients are, however, rare. Most have heretofore focused on cells generated from monocytes following *ex vivo* culture with GM-CSF and IL-4 (mo-DCs) [14]. These express reduced surface MHC II expression compared with cells from healthy individuals, but no alteration in production of IL-23 or other cytokines [15, 16]. On the other hand, *in vitro* studies do suggest that ER stress of DCs could lead to excessive IL-23 production [17], and as such they are intriguing candidate cells in initiating this effector pathway. In this regard, however, the relevance of data generated using cultured monocytes to DC biology is unclear [18]. We consider it critical now to determine the contributions of bone fide DCs and other myeloid lineages to AS pathogenesis.

Our previous analyses revealed a systemic deficiency in a specific DC population in B27-TG rats, which in turn promoted elaboration of Th17 responses [19]. Because this cytokine axis may be associated with AS pathology, we hypothesized that myeloid populations could be similarly altered in AS patients. Accordingly, we performed deep phenotyping of blood and SF leucocyte populations and now show that the frequency of circulating CD1c^+^ DCs is diminished in AS patients. Conversely, proportions of CD14^−^ CD16^+^ mononuclear cells are elevated and functionally promote CCR6 induction and IL-6 and IL-1β secretion following T cell interaction. Furthermore, we identify a correlation between systemic inflammation and a gut-homing phenotype among T cells from AS patients. Thus, we provide novel insight into the pathways that can promote chronic inflammation mediated through the Th17 axis.

## Materials and methods

### Patients

AS patients meeting the modified New York criteria [20] were recruited from the Glasgow Royal Infirmary rheumatology clinic between March 2011 and June 2013. Informed, written consent was obtained from all study participants according to the Declaration of Helsinki. Ethical approval for the study was awarded by the West of Scotland Research Ethics Service (Institute of Infection, Immunity and Inflammation Research Tissue Bank, REC: 11/S0704/7). Consenting age- and sex-matched healthy controls (HCs) were recruited under ethics approved by the College of Medical, Veterinary, and Life Sciences Ethics Committee, University of Glasgow (Project #2013007). Patient clinical features are outlined in supplementary Table S1, available at *Rheumatology* Online.

### Reagents

Cells were cultured in RPMI 1640 supplemented with 10% fetal calf serum, 100 U/ml penicillin, 100 μg/ml streptomycin, 2 mM l-glutamine and 50 μM 2-mercaptoethanol (complete medium).

### Peripheral blood mononuclear cell isolation

Isolation of peripheral blood mononuclear cells was performed over a Histopaque–1077 gradient. Following centrifugation, the peripheral blood mononuclear cell layer was harvested.

### Antibodies

Directly conjugated or biotin-labelled antibodies targeting CD3 (UCHT-1), CD4 (OKT4), CD14 (M5E2), CD15 (W6D3), CD16 (3G8), CD19 (HIB19), CD25 (BC96), CD45RA (HI100), CD56 (MEM-188), HLA-DR (L243), TcRαβ (IP26) and CXCR3 (GO25H7) were from Biolegend (San Diego, CA, USA). Antibodies targeting CCR9 (248621) and CCR10 (314305) were purchased from R&D systems (Minneapolis, MN, USA). CD1c (AD5-8E7) and anti-SLAN (M-DC8, DD-1) antibodies were from Miltenyi Biotec (Auburn, CA, USA). CD11c (B-ly6), CD141 (1A4), CCR6 (11A9) and CCR4 (1G1) antibodies were purchased from BD Biosciences (Oxford, UK).

### Flow cytometry

Following Fc receptor inhibition (eBioscience, San Diego, CA, USA), staining was performed in PBS with 2% fetal calf serum and 2 mM EDTA. Where biotin-conjugated antibodies were used, a streptavidin step was performed. Samples were acquired using LSR II (BD Biosciences) or MACSQuant (Miltenyi Biotec) flow cytometers, or purified using a FACSAria cell sorter (BD Biosciences). Data were analysed using FlowJo software (version 9.2; Tree Star, Ashland, OR, USA).

### Naive CD4^+^ T cell isolation

Naive CD4^+^ T cells were isolated from peripheral blood mononuclear cells using a naive CD4^+^ T cell isolation kit (Miltenyi Biotec). Cells were stained with carboxyfluorescein succinimidyl ester (CFSE: 5 μM).

### Mixed leucocyte reaction

Myeloid populations were resuspended in complete medium and co-cultured with CFSE-labelled allogeneic naive CD4^+^ T cells for 5 days. Supernatants were harvested and proliferation assessed through CFSE dilution.

### RNA extraction

A MicroRNA kit (Qiagen, Venlo, Netherlands) was used for RNA extraction. Genomic DNA was removed using an RNase-free DNase kit (Qiagen). cDNA was generated using a Superscript First Strand Kit (Invitrogen).

### Real-time quantitative PCR

Gene expression was measured using the Brilliant III Ultra Fast SYBR GREEN qRT-PCR Master Mix (Agilent Technologies, Santa Clara, CA, USA). Reactions were analysed using a 7500 Fast Real-Time PCR System machine (Applied Biosystems, Foster City, CA, USA). Primers used were TATA-binding protein (TBP) : forward AGACCTTCCTGTTTACCCTTG, reverse TAGCTGTGGGTGACTGCTTGG; ATF4: forward GACCACGTTGGATGACACTTG, reverse GGGAAGAGGTTGTAAGAAGGTG; PERK: forward TGCCTGGCTCGAAGCACCAC, reverse TGGTGCATCCATTGGGCTAGGA; EIF2α: forward GCTCTTGACAGTCCGAGGAT, reverse CATTGCCCCAGGCAAACAAG; unspliced XBP-1: forward AGACAGCGCTTGGGGATGGAT, reverse CCTGCTGCAGAGGTGCACGTAG; spliced XBP-1: forward AGACAGCGCTTGGGGATGGAT, reverse CCTGCACCTGCTGCGGACTC; ATF6: forward TCAGACAGTACCAACGCTTATGC, reverse GTTGTACCACAGTAGGCTGAGA; and Bip: forward TGCTTGATGTATGTCCCCTTA, reverse CCTTGTCTTCAGCTGTCACT. Expression levels were normalized to TBP. Results were presented as 2^−^^ΔΔCt^.

### Cytokine detection

Cytokines IL-1β, IL-4, IL-5, IL-6, IL-10, IL-17A, GM-CSF, IFNγ and TNFα were detected using a custom-made Luminex assay (R&D systems), using the BioPlex 200 system (Bio-Rad, Hercules, CA, USA). Flt3L, IL-23p19 and IL-12p70 were detected by ELISA (R&D systems). Plates were read using an MRX microplate reader (Dyn-ex technologies, Chantilly, VA, USA).

### Statistics

Results were presented as +s.d. Data were analysed by Student’s *t*-test or Mann–Whitney *U* test, followed by a Bonferroni post-test using GraphPad prism (San Diego, CA, USA). Linear regression, Kruskal–Wallis Spearman correlative tests were performed with Dunn multiple comparisons post-tests. Values of *P* < 0.05 were considered statistically significant.

## Results

### The CD4^+^ T cell CCR6/CXCR3 axis is altered in AS patients

**Fig. 1:**
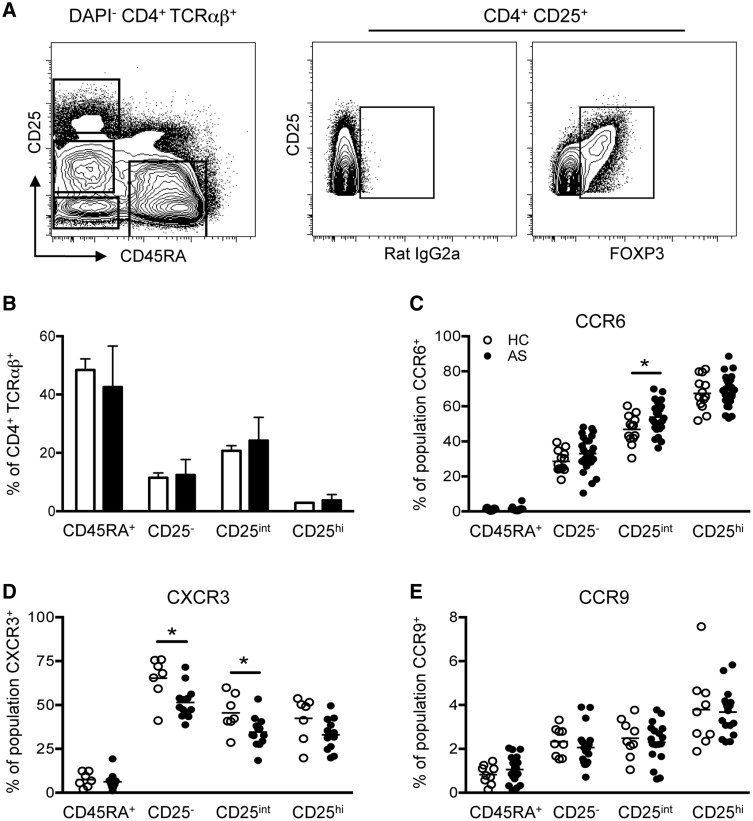
Expression of CCR6 and CXCR3 on circulating CD4^+^ T cell subsets (**A**) CD25 and CD45RA define four circulating CD4^+^ T cell populations. FOXP3 expression on total CD25^+^ cells. (**B**) Quantification of naive (CD45RA^+^), memory (CD25^−^), activated (CD25^int^) and regulatory (CD25^hi^) T cells in HCs (*n* = 14, empty bars) and AS patients (*n* = 27, filled bars). CCR6 (**C**), CXCR3 (**D**) and CCR9 (**E**) expression on CD4^+^ T cell subsets [HCs (empty circles), *n* = 7–13; AS patients (filled circles), *n* = 13–26]. Each dot represents one individual. *P < 0.05, Mann–Whitney test performed for CXCR3 expression on CD25^−^ T cells, otherwise unpaired Student’s tests were performed. Error bars show mean + s.d.

T cells belonging to the Th17 lineage have been associated with disease pathogenesis in B27-TG rats [9, 19] and AS patients [10–12]. We performed detailed immunophenotyping of the circulating CD4^+^ T cell profile in AS through quantification of T cell populations and their effector status. The latter aspect was determined through examination of chemokine receptor expression (commonly used surrogate markers of T helper cell polarization) [21, 22]. We first segregated four CD4^+^ TcRαβ^+^ populations based on CD25 and CD45RA expression (Fig. 1A), namely naive (CD45RA^+^ CD25^−^), memory (CD45RA^−^ CD25^−^), activated (CD45RA^−^ CD25^int^) phenotypes and Tregs (CD45RA^−^ CD25^hi^). All Tregs expressed FoxP3 (Fig. 1A). Enumeration of CD4^+^ T cell subsets revealed no differences between AS patients and HCs (Fig. 1B). Elevated levels of CCR6 and reduced CXCR3 expression were observed on activated (CD25^int^) CD4^+^ AS patient T cells (Fig. 1C and D). CXCR3 expression was also reduced on AS CD4^+^ memory T cells (Fig. 1D). CCR9, CCR4 and CCR10 were not differentially expressed on HC and AS patient CD4^+^ T cell populations (Fig. 1E and supplementary Fig. S1, available at *Rheumatology* Online). These data highlight differences in the chemokine receptor profile of circulating CD4^+^ T cells in AS patients, with a propensity towards elevated proportions of CCR6-expressing effector populations.

### AS patient plasma contains elevated levels of Th17-associated cytokines

**Fig. 2:**
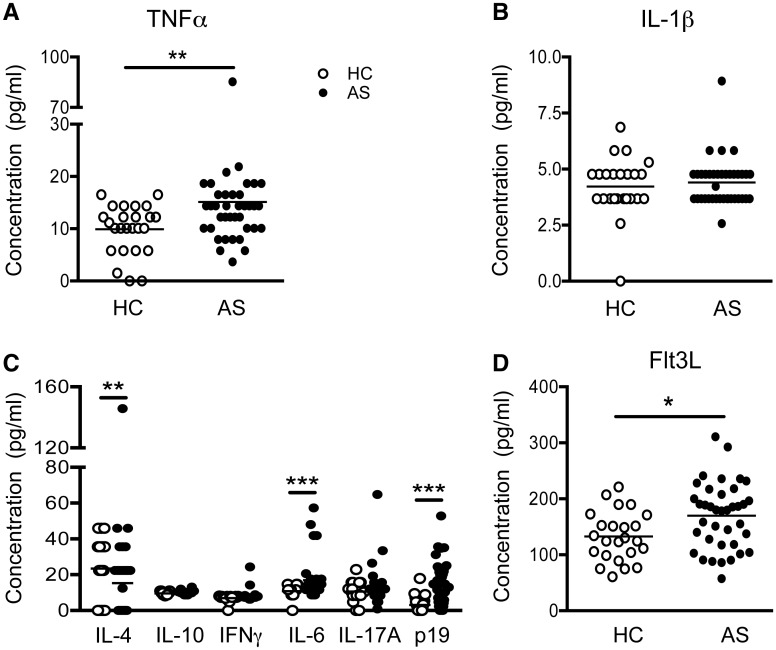
Systemic cytokine profile of AS patients Cytokine and growth factor levels were determined by Luminex or ELISA for HCs (empty circles) and AS patients (filled circles). Levels of TNFα (**A**) and IL-1β (**B**) in HC (*n* = 24–25) and AS patient (*n* = 38) plasma. (**C**) Detection of Th cell-associated cytokines IL-4, IL-10, IFNγ, IL-6, IL-17A and IL-23p19 within HC (*n* = 23–24) and AS patient (*n* = 38) plasma. (**D**) Amount of plasma Flt3L in HCs (*n* = 24) and AS patients (*n* = 38). Statistical analyses were performed between HCs and AS patients for each parameter. *P < 0.05, **P < 0.01, ***P < 0.001 using Mann–Whitney tests.

To understand how the balance of T helper subsets might be regulated in AS patients, we examined the plasma cytokine and growth factor profiles in our cohort. We observed a modest increase in TNFα levels in AS patient plasma (Fig. 2A). IL-1β levels were similar to those of controls (Fig. 2B). Assessment of several plasma Th-associated cytokines revealed a significant reduction in plasma IL-4 in AS patients, while IL-10 and IFNγ levels resembled those of HCs (Fig. 2C). In contrast, there was significant upregulation in the circulating levels of the Th17-associated cytokines IL-6 and IL-23p19, but no increase in IL-17A itself (Fig. 2C). Thus, the cytokine milieu in AS patients is skewed towards the Th-17/IL-23 axis. Interestingly, Flt3L, a myeloid haematopoietic growth factor, was significantly upregulated in AS patient plasma compared with HCs (Fig. 2D).

### The proportion of circulating CD16^+^ mononuclear cells increased in AS

**Fig. 3:**
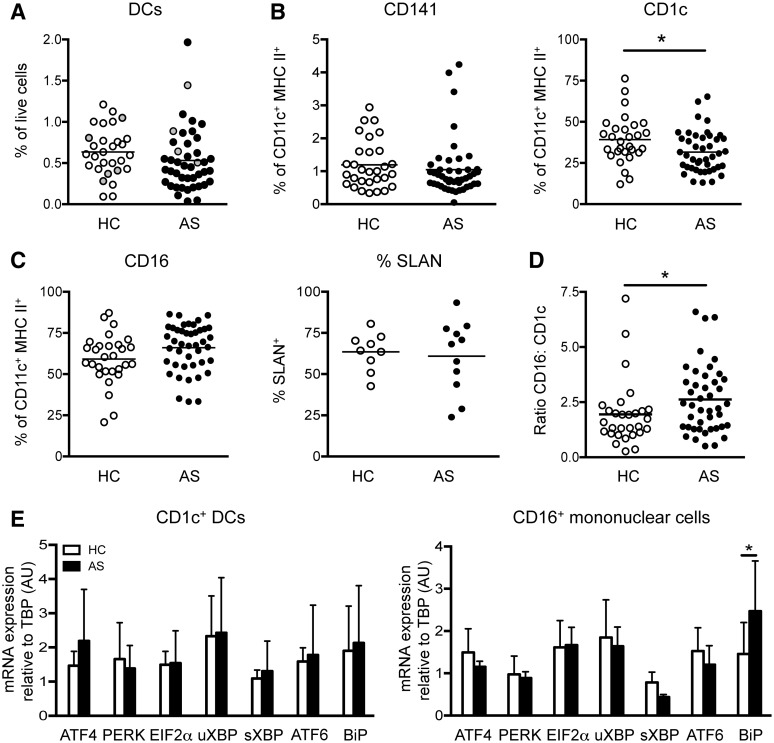
Circulating myeloid profile of AS patients (**A**) Enumeration of total circulating HC (*n* = 29) and AS patient (*n* = 43) DCs. Grey dots indicate HLA-B27^+^ HCs and HLA-B27^−^ AS patients where appropriate. CD141^+^ and CD1c^+^ DCs (**B**), total CD16^+^ and SLAN^+^ CD16^+^ mononuclear cells (**C**) as a proportion of LIN^−^ (CD3/CD15/CD19/CD56) CD14^−^ CD11c^+^ HLA-DR^+^ cells in HCs (*n* = 9–30) and AS patients (*n* = 11–43). (**D**) Ratio of CD16^+^ mononuclear cells:CD1c^+^ DCs in HCs (*n* = 29) and AS patients (*n* = 43). (**E**) ER stress gene expression in FACS-purified HC (*n* = 4) and AS patient (*n* = 3) myeloid cells, relative to TATA-binding protein (TBP) using the 2^−ΔΔCt^ method with the HC population equal to 1 (arbitrary units. *P < 0.05, Mann–Whitney test or two-way ANOVA. Error bars show ± s.d.

Data from our laboratory support a relationship between systemic myeloid dysregulation and AS/SpA; we previously demonstrated that B27-TG rats lack CD103^+^ CD172a^lo^ DCs [19], while Flt3L was significantly upregulated in AS patients (Fig. 2D). Thorough examination of myeloid populations may elucidate their involvement in promoting the aberrant T cell phenotypes observed in our patient cohort. To our knowledge, no prior study has directly addressed this question *ex vivo* without prior cell expansion in culture. Lineage^−^ CD14^−^ CD11c^+^ HLA-DR^+^ cells were subdivided into three myeloid populations based on their differential expression of CD141 and CD16 (supplementary Fig. S2A and B, available at *Rheumatology* Online). The CD141^−^ CD16^−^ population expresses CD1c (data not shown) and thus represents CD1c^+^ DCs. The CD141^−^ CD16^+^ population [23], collectively termed CD14^−^ CD16^+^ mononuclear cells, was heterogeneous for 6-Sulpho LacNAc (SLAN) (M-DC8) and thus contained CD14^−^ CD16^+^ SLAN^−^ and SLAN^+^ subsets (supplementary Fig. S2A, available at *Rheumatology* Online). No difference was observed in the proportion of total DCs (CD1c^+^ and CD141^+^ DCs) between HCs and AS patients (Fig. 3A). Unfortunately, our study was insufficiently powered, with too few HLA-B27^+^ HCs and HLA-B27^−^ AS patients to examine the influence of HLA-B27 on immunological disease parameters (Fig. 3A). Analysis of plasmacytoid DCs (HLA-DR^+^ CD123^+^ CD304^+^) revealed no significant difference between HCs and AS patients (supplementary Fig. S2C, available at *Rheumatology* Online). We observed no disparities in the frequencies of CD141^+^ DCs in AS patients (Fig. 3B). However, analogous to that population we predicted would be deficient from our B27-TG experiments [19], analysis of clinical data (supplementary Table S1, available at *Rheumatology* Online) revealed a significant correlation between increased proportions of CD141^+^ DCs and greater disease severity when assessed by BASDAI (disease activity) but not by BASFI (for functional limitation see supplementary Fig. S3, available at *Rheumatology* Online).

Unexpectedly, we observed a significant proportional reduction in CD1c^+^ DCs in AS patients (Fig. 3B). This finding was offset by increased frequencies of total CD14^−^ CD16^+^ mononuclear cells in AS patients (*P* = 0.0552), despite no proportional differences in SLAN-expressing subsets (Fig. 3C). Comparison of the major blood myeloid populations revealed a significant shift from CD1c^+^ DCs towards CD14^−^ CD16^+^ mononuclear cells in AS patients (Fig. 3D). The unfolded protein response (UPR) induced within myeloid cells as a consequence of ER stress initiated through HLA-B27 misfolding, is thought to contribute to disease pathogenesis in B27-TG rats [7–9]. Despite the observed myeloid dysregulation (Fig. 3B and C), we failed to detect UPR induction in AS patient CD1c^+^ DCs and CD14^−^ CD16^+^ mononuclear cells (Fig. 3E). Upregulation of BiP expression was observed within CD14^−^ CD16^+^ mononuclear cells (Fig. 3E).

### Myeloid populations can be identified in AS patient SF

**Fig. 4:**
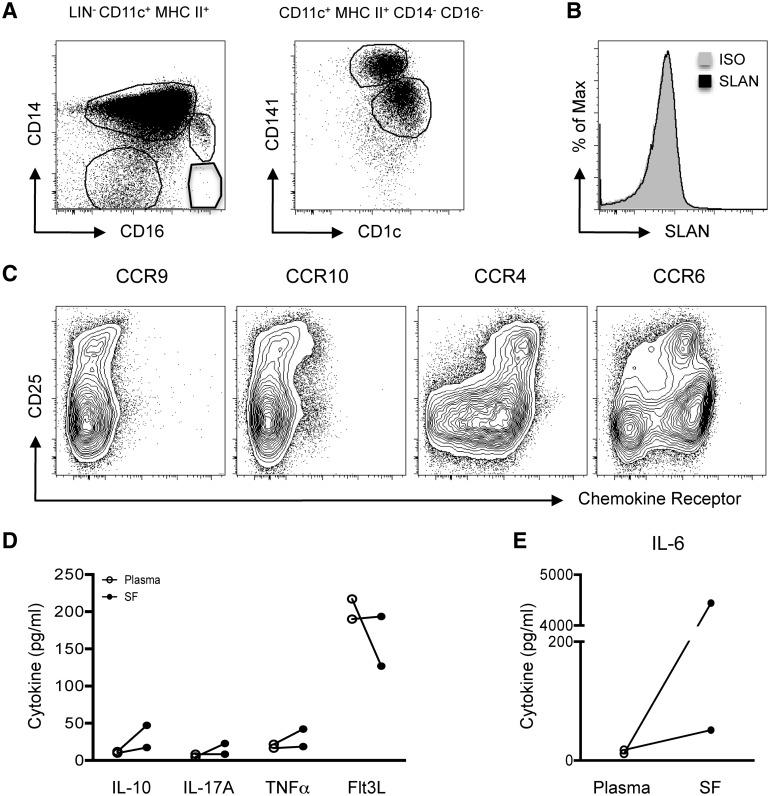
Immunological milieu of AS SF (**A**) Live, single LIN^−^ (CD3/CD15/CD19/CD56) CD11c^+^ HLA-DR^+^ cells were divided into three myeloid subsets: CD14^+^ CD16^−^ monocytes, CD14^+^ CD16^+^ monocytes and CD14^−^ CD16^−^ cells. The CD14^−^ CD16^−^ subset contained SF DCs—CD141^+^ and CD1c^+^ populations. (**B**) SLAN expression on total live LIN^−^ CD14^−^ CD11c^+^ HLA-DR^+^ cells, compared with isotype (shaded grey). (**C**) CCR9, CCR10, CCR4 and CCR6 expression on AS patient (*n* = 1) CD3^+^ CD4^+^ SF T cells. AS patient (*n* = 2) concentrations of IL-10, IL-17A, TNFα, Flt3L (**D**) and IL-6 (**E**) in matched plasma (empty) and SF (filled).

A presumed pathophysiological destination of circulating cells is the articular compartment. As tissue is not readily available from the spine, we analysed the immune cell milieu of four AS patient SF samples. Analysis of lineage^−^ CD11c^+^ MHC II^+^ cells revealed four myeloid populations: CD14^+^ CD16^−^ monocytes, CD14^+^ CD16^+^ monocytes and two DC populations (CD141^+^ and CD1c^+^; Fig. 4A). In contrast to peripheral blood (Fig. 3C and supplementary Fig. S2B, available at *Rheumatology* Online), the CD14^−^ CD16^+^ SLAN^+^ and SLAN^−^ mononuclear cell subsets were absent from AS SF (Fig. 4A and B). High levels of CCR4 and CCR6 expression were observed on SF CD3^+^ CD4^+^ T cells (Fig. 4C), and most CD4^+^ CD25^hi^ cells co-expressed both chemokine receptors (Fig. 4C). To identify inflammatory mediators driving joint pathology, the cytokine milieu from matched AS patient SF and blood samples was analysed. IL-10, IL-17A, TNFα and Flt3L were not differentially expressed between plasma and SF (Fig. 4D). In contrast, IL-6 concentrations were dramatically increased within AS SF (Fig. 4E).

### DC–T cell interactions promote Th17-like immune responses

**Fig. 5:**
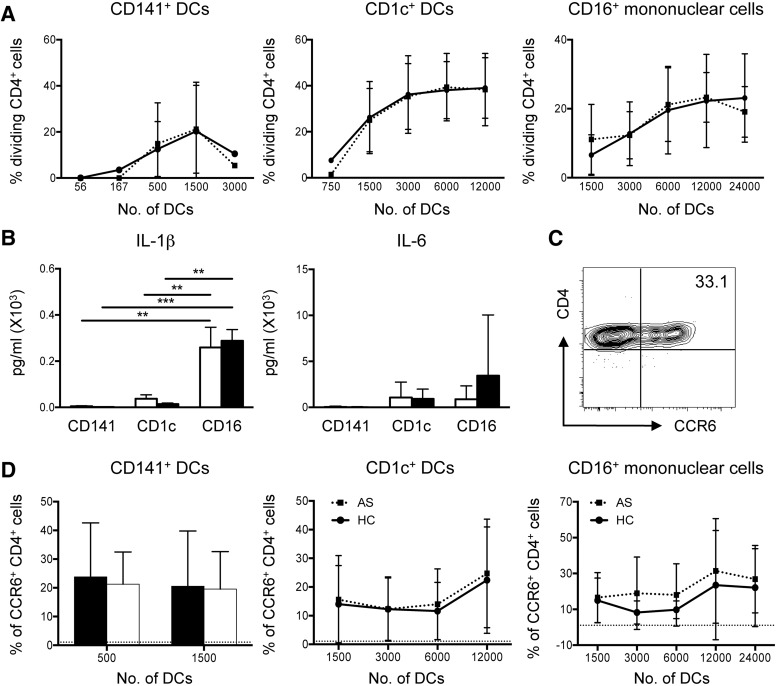
Functional assessment of DC : T cell interactions in AS (**A**) CD4^+^ T cell proliferation assessed by mixed leucocyte reaction (MLR) with indicated myeloid populations. (**B**) IL-1β and IL-6 secretion following MLR. (**C**) Representative plot depicting CCR6 induction on proliferating CD4^+^ T cells following HC CD1c^+^ DC co-culture. (**D**) Quantification of CCR6 expression on proliferating CD4^+^ T cells following MLR with individual myeloid subsets. For analysis, 4–10 HCs and 6–15 AS patients were used. Dotted line at 1.078 equates to level of CCR6 expression on naive CD4^+^ T cells. **P < 0.01 and ***P < 0.001. Mann–Whitney test or two-way ANOVA (**B**) were performed. Error bars show ± s.d.

Cognate interactions between antigen-presenting cells, principally DCs, and T cells are central to the regulation of immune responses. To investigate blood myeloid cell function, we assessed their ability to stimulate proliferation of naive allogeneic CD4^+^ T cells. HC and AS patient myeloid cells induced similar CD4^+^ T cell proliferation at all DC:T cell ratios (Fig. 5A). DCs have a greater capacity to induce naive T cell proliferation than blood monocytes (supplementary Fig. S4, available at *Rheumatology* Online). Supernatant analysis from these co-cultures revealed no difference in IL-1β and IL-6 production between HCs and AS patients (Fig. 5B). CD14^−^ CD16^+^ mononuclear cell–CD4^+^ T cell co-cultures consistently secreted the highest levels of IL-1β and IL-6 (Fig. 5B). The Th-associated cytokines IFNγ and IL-17A were also examined; however, no significant differences were observed (data not shown). Overall, interactions between CD14^−^ CD16^+^ mononuclear cells and T cells promote secretion of the Th17-associated cytokines IL-1β and IL-6.

CCR6 is often used as a surrogate marker for Th17 cells [22], although not all CCR6^+^ cells secrete IL-17A (data not shown). Given that published data implicate Th17 cell involvement in AS pathogenesis [10–12], and the elevated CCR6 expression on AS patient CD4^+^-activated T cells observed by us, we explored whether these phenomena could arise from altered DC–T cell interactions. We performed mixed leucocyte reactions and assessed CCR6 expression on proliferating CD4^+^ T cells (Fig. 5C). Blood DCs and CD14^−^ CD16^+^ mononuclear cells from AS patients and HCs all induced CCR6 on interacting T cells, to similar levels (Fig. 5D).

### Disease pathogenesis linked to CCR6^+^ and CCR9^+^ CD4^+^ T cells

**Fig. 6:**
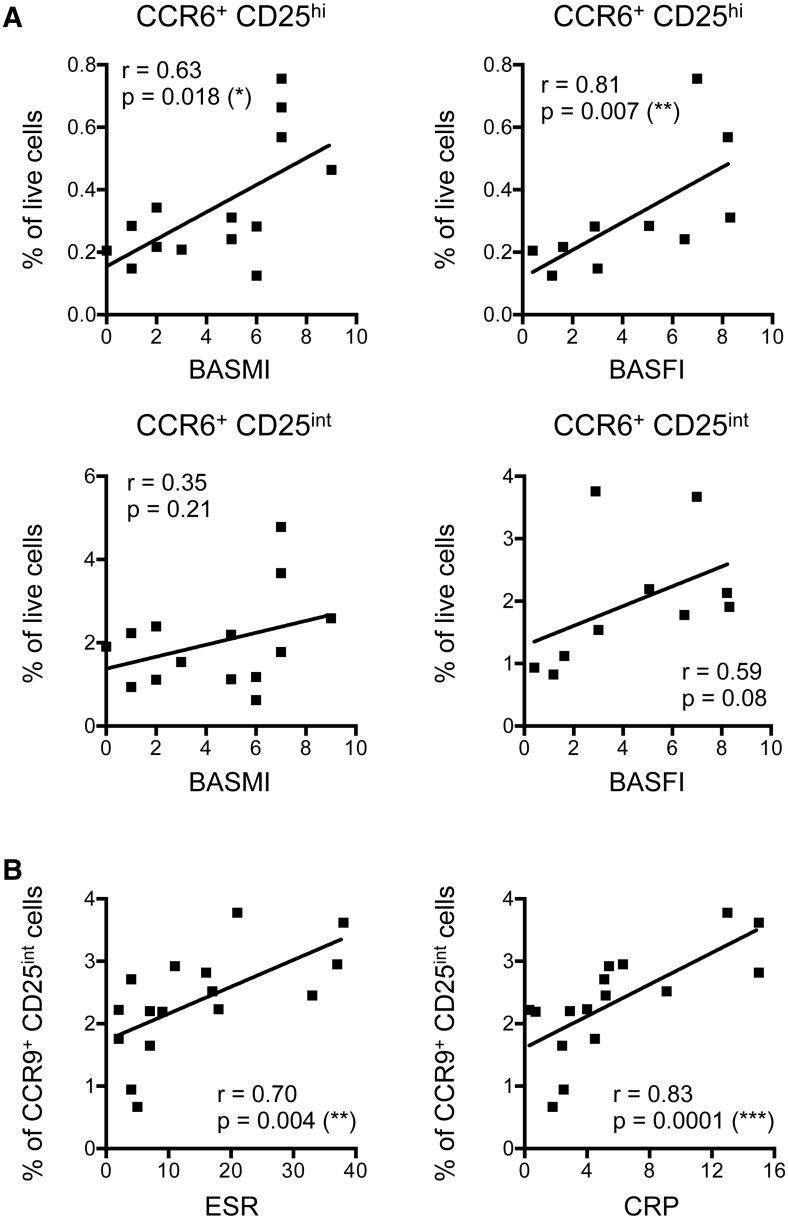
Immunological parameter correlations with disease progression and inflammation in AS (**A**) Correlations between AS patient (*n* = 14) CCR6-expressing CD4 ^+ ^CD25^hi^ T cells (Tregs; Top) and CCR6^+^ CD25^int^ T cells (activated; bottom) with BASMI (left) and BASFI (right) clinical scores. (**B**) ESR and CRP correlations with AS patient (*n* = 16) CCR9^+^ CD4^+^ CD25^int^ (activated) cell frequencies. Each dot represents one individual. Analyses were performed and assessed using linear regression and the Kruskal–Wallis Spearman correlative statistical tests (*r*), using the Dunn multiple comparisons post-test. *P < 0.05, **P < 0.01 and ***P < 0.001.

Finally, to further explore our data relative to clinical phenotypes, we evaluated the relationships between immunological and clinical parameters. We observed that increased frequencies of CCR6^+^ Tregs (CD25^hi^) were significantly associated with elevated disease severity, assessed by both BASMI and BASFI (Fig. 6A). Although similar relationships were observed between increasing frequencies of CCR6^+^ activated T cells (CD25^int^) and BASMI and BASFI, these did not reach significance (Fig. 6A). Furthermore, we observed significant correlations between increased proportions of CCR9^+^ CD4^+^ activated T cells and elevated ESR and CRP scores (Fig. 6B).

## Discussion

The genetic link between HLA-B27 and AS was identified more than 40 years ago, yet the mechanisms underpinning this association remain elusive. Studies of B27-TG rats indicate involvement of myeloid populations and CD4^+^ T cells (specifically the Th17 lineage) in SpA pathology [5–9, 19, 24]. Parallel human studies have highlighted involvement of the Th17/IL-23 pathway in AS [1, 2, 12, 25]. Despite this, evidence for IL-17–secreting T cells in inflamed, extra-articular tissues is limited [26–28]. Furthermore, examination of the critical myeloid populations has only been performed indirectly, using *in vitro*-generated mo-DCs [15, 16]. These cultures do not recapitulate the diverse populations found *in vivo*. We have performed the first detailed phenotypic and functional analyses of freshly isolated myeloid populations in AS. We have not, however, performed these analyses using samples from patients with other inflammatory diseases. Such comparisons have previously been shown to be valuable [29, 30]. Further studies are therefore required to establish whether our immunological observations are unique to AS pathophysiology. Additionally, the majority of our patients presented with long-standing disease; if samples were available it would be fascinating to repeat these studies using patients in the earliest stages of disease.

Significantly, our analyses support reports of a systemic imbalance in circulating CD4^+^ T cell subsets in AS, favouring cells of the Th17 lineage. We identified this imbalance using CCR6 and CXCR3 as surrogate markers of Th17 and Th1 differentiation, respectively [21, 22]. The elevations we observe in circulating levels of the Th17-associated cytokines IL-6 and IL-23 may promote induction and survival of these Th17-like cells *in vivo* [31, 32]. IL-23 has also been shown to directly drive SpA-associated pathology in animal models, and has been associated with human disease [26, 33, 34]. Our data support these observations, implicating involvement of CCR6^+^/Th17-associated CD4^+^ lymphocytes and the IL-23 cytokine axis in AS pathogenesis [9, 19, 25].

We previously identified a systemic deficiency in the CD103^+^ CD172a^lo^ tolerogenic DC population in B27-TG rats [19]. We hypothesized that the analogous human subset, CD141^+^ DCs, would be absent or depleted in AS patients. However, we observed no differences in the frequencies of circulating CD141^+^ DCs between AS patients and HCs. In fact, elevated proportions of circulating CD141^+^ DCs were weakly associated with increased disease severity. Thus, unlike in B27-TG rats, a loss of this DC subset does not appear to drive human disease. Hence, several immunological processes driving human AS appear to differ from those driving SpA-like symptoms in B27-TG rats. In contrast, we observed a significant reduction in the frequency of the largest blood DC population, CD1c^+^ DCs, in AS patients. It should be noted that there was a 15-year difference between the average ages of the HC and AS patient cohorts. We consider it unlikely that this disparity caused the observed differences between our HC and AS patient populations because our analyses did not detect any influence of donor age on the number or frequency of any DC population in our cohorts (data not shown). In myeloid cells of B27-TG rats, the UPR, driven by misfolded HLA-B27 molecules, induces release of pro-inflammatory cytokines and cell death [9, 35, 36]. However, we could detect no evidence for UPR activation in circulating CD1c^+^ DCs and CD14^−^ CD16^+^ mononuclear cells from AS patients. Our results thus support previous observations using more heterogeneous cell preparations from blood or intestine [37–41] and implicate involvement of alternative disease-associated immunopathogenic mechanisms. If HLA-B27–induced UPR induction is indeed the force driving AS, future investigations will need to carefully examine whether any tissue-resident cell populations show evidence of ER stress or UPR activation.

Accompanying the reduced frequency of blood CD1c^+^ DCs was an increase in the heterogeneous CD14^−^ CD16^+^ mononuclear cell population. This population contains both patrolling CD16^+^ blood monocytes [23] and M-DC8/SLAN^+^ DCs [42, 43]. To understand the functional consequences of these changes, we assessed the ability of these myeloid populations to activate naive T cells. On a per-cell basis, no differences were observed between AS patient and HC myeloid cells; CD14^−^ CD16^+^ mononuclear cells promoted secretion of the Th17-associated cytokines IL-1β and IL-6 and were capable of inducing CCR6 expression on interacting T cells. Consequently, the observed shift towards the CD14^−^ CD16^+^ myeloid population in AS patients may contribute to the induction of the pathology-associated Th17 response. Of note, SpA patient CD1c^+^ DCs express elevated levels of CD1d, which was shown to have immunoregulatory properties [44]. Our observation, that there is a reduction in the frequency of circulating CD1c^+^ DCs in AS patients, contributes to the evidence suggesting that myeloid cells may be involved with disease progression. To test this connection, further studies are required to address the role of DCs in driving disease at sites of ongoing inflammation in AS.

Analyses of the more readily available cells in blood may not accurately reflect immunological processes occurring in disease-affected tissues. Examination of tissue populations can therefore be helpful in elucidating pathogenic mechanisms. SF samples from peripheral joints, although not necessarily representative of AS spinal or musculoskeletal pathophysiology, remain an important resource for characterizing potential tissue effector populations. We identified CD1c^+^ DCs in AS SF, as previously observed in RA SF [45]. Additionally, we identified a high frequency of CD141^+^ DCs in AS SF. These SF-resident DCs may perpetuate disease by driving local T cell responses. However, because these results were generated using very limited numbers of samples, further phenotypic and functional analyses must be performed to better understand the role of the immune populations residing within the SF. Of note, CD14^−^ CD16^+^ mononuclear cells were completely absent from AS SF, indicating that they do not act locally to drive peripheral joint disease in AS. CD14^−^ CD16^+^ SLAN^+^ cells have, however, been identified in inflamed extra-articular tissues, including tonsil and psoriatic skin [46, 47]. Together with our results, these data suggest that extra-articular CD14^−^ CD16^+^ cells in lymphoid or other peripheral tissues may contribute to AS pathogenesis.

To understand how immunological changes may contribute to disease development, we must consider how these differences relate to patient clinical characteristics. In our cohort, it was clear that CCR6^+^ Tregs were more frequent in patients with the highest disease scores. From this correlation we infer that in AS patients, CCR6-expressing Tregs may be induced in an attempt to suppress pathology. Further investigation is clearly required to accurately assess whether this population has any effect on disease progression.

Interestingly, our data indicate a relationship between intestinal immune responses and systemic inflammation in AS; elevated ESR and CRP values correlate with higher proportions of circulating CCR9-expressing activated T cells. CCR9 is induced on T cells activated in intestinal lymphoid tissues [48]. Thus, this correlation indicates that systemic inflammation in AS is associated with intestinal activation of T cells. This is potentially extremely important, given the absence of inflammation from B27-TG rats housed under germ-free conditions, and the fact that ∼50% of AS patients show evidence of subclinical intestinal inflammation [49, 50]. Together, these results indicate that antigen-presenting cells of intestinal origin may play a central role in driving systemic inflammation in AS patients. Given this association between systemic inflammation and the intestinal immune response, it is important to understand the impact of peripheral disease manifestations, especially signs of IBD, on disease and immunological parameters. Unfortunately, our study was not sufficiently powered (containing too few patients with IBD) to address this directly.

In summary, we have performed extensive immunophenotyping of AS patients and have identified disease-associated alterations in circulating T lymphocytes and myeloid populations. Specifically, elevated proportions of CD14^−^ CD16^+^ mononuclear cells, capable of inducing CCR6-expressing T cells and IL-1β and IL-6 production, may contribute to the Th17 immune responses that are associated with AS. Furthermore, intestinal immune responses appear to contribute to systemic inflammation in AS patients, implicating loss of intestinal homeostasis as an important factor driving disease. Together, these observations enhance our understanding of the pathogenesis of AS, and support the idea that aberrant T cell responses and intestinal inflammation contribute to the pathogenesis of this disease.

## Supplementary Material

Supplementary Data

## References

[1] Australo-Anglo-American Spondyloarthritis Consortium (TASC): Reveille JD Sims A-M Danoy P Evans DM Leo P Genome-wide association study of ankylosing spondylitis identifies non-MHC susceptibility loci. Nat Genet 2010;42:123–7. 20062062 10.1038/ng.513 PMC3224997

[2] Wellcome Trust Case Control Consortium, Australo-Anglo-American Spondylitis Consortium [TASC]: Burton PR Clayton DG Cardon LR Craddock N Deloukas P Association scan of 14,500 nonsynonymous SNPs in four diseases identifies autoimmunity variants. Nat Genet 2007;39:1329–37. 17952073 10.1038/ng.2007.17 PMC2680141

[3] Danoy P Pryce K Hadler J Association of variants at 1q32 and STAT3 with ankylosing spondylitis suggests genetic overlap with Crohn’s disease. PLoS Genet 2010;6:e1001195. 21152001 10.1371/journal.pgen.1001195 PMC2996314

[4] Baeten D Baraliakos X Braun J Anti-interleukin-17A monoclonal antibody secukinumab in treatment of ankylosing spondylitis: a randomised, double-blind, placebo-controlled trial. Lancet 2013;382:1705–13. 24035250 10.1016/S0140-6736(13)61134-4

[5] Breban M Fernández-Sueiro JL Richardson JA T cells, but not thymic exposure to HLA-B27, are required for the inflammatory disease of HLA-B27 transgenic rats. J Immunol 1996;156:794–803. 8543835

[6] May E Dorris ML Satumtira N CD8 alpha beta T cells are not essential to the pathogenesis of arthritis or colitis in HLA-B27 transgenic rats. J Immunol 2003;170:1099–105. 12517979 10.4049/jimmunol.170.2.1099

[7] Hoentjen F Tonkonogy SL Liu B Adoptive transfer of nontransgenic mesenteric lymph node cells induces colitis in athymic HLA-B27 transgenic nude rats. Clin Exp Immunol 2006;143:474–83. 16487247 10.1111/j.1365-2249.2006.03013.x PMC1809603

[8] Breban M Hammer RE Richardson JA Taurog JD Transfer of the inflammatory disease of HLA-B27 transgenic rats by bone marrow engraftment. J Exp Med 1993;178:1607–16. 8228809 10.1084/jem.178.5.1607 PMC2191228

[9] DeLay ML Turner MJ Klenk EI HLA-B27 misfolding and the unfolded protein response augment interleukin-23 production and are associated with Th17 activation in transgenic rats. Arthritis Rheum 2009;60:2633–43. 19714651 10.1002/art.24763 PMC2893331

[10] Shen H Goodall JC Hill Gaston JS Frequency and phenotype of peripheral blood Th17 cells in ankylosing spondylitis and rheumatoid arthritis. Arthritis Rheum 2009;60:1647–56. 19479869 10.1002/art.24568

[11] Limón-Camacho L Vargas-Rojas MI Vázquez-Mellado J *In vivo* peripheral blood proinflammatory T cells in patients with ankylosing spondylitis. J Rheumatol 2012;39:830–5. 22337239 10.3899/jrheum.110862

[12] Zhang L Li Y-G Li Y-H Increased frequencies of Th22 cells as well as Th17 cells in the peripheral blood of patients with ankylosing spondylitis and rheumatoid arthritis. PLoS ONE 2012;7:e31000. 22485125 10.1371/journal.pone.0031000 PMC3317658

[13] Fert I Glatigny S Poulain C Correlation between dendritic cell functional defect and spondylarthritis phenotypes in HLA-B27/HUMAN beta2-microglobulin-transgenic rat lines. Arthritis Rheum 2008;58:3425–9. 18975325 10.1002/art.24023

[14] Xu Y Zhan Y Lew AM Naik SH Kershaw MH Differential development of murine dendritic cells by GM-CSF versus Flt3 ligand has implications for inflammation and trafficking. J Immunol 2007;179:7577–84. 18025203 10.4049/jimmunol.179.11.7577

[15] Slobodin G Kessel A Kofman N Phenotype of resting and activated monocyte-derived dendritic cells grown from peripheral blood of patients with ankylosing spondylitis. Inflammation 2012;35:772–5. 21833763 10.1007/s10753-011-9373-x

[16] Prevosto C Goodall JC Hill Gaston JS Cytokine secretion by pathogen recognition receptor-stimulated dendritic cells in rheumatoid arthritis and ankylosing spondylitis. J Rheumatol 2012;39:1918–28. 22896020 10.3899/jrheum.120208

[17] Goodall JC Wu C Zhang Y Endoplasmic reticulum stress-induced transcription factor, CHOP, is crucial for dendritic cell IL-23 expression. Proc Natl Acad Sci U S A 2010;107:17698–703. 20876114 10.1073/pnas.1011736107 PMC2955096

[18] Bain CC Scott CL Uronen-Hansson H Resident and pro-inflammatory macrophages in the colon represent alternative context-dependent fates of the same Ly6C[hi] monocyte precursors. Mucosal Immunol 2013;6:498–510. 22990622 10.1038/mi.2012.89 PMC3629381

[19] Utriainen L Firmin D Wright P Expression of HLA-B27 causes loss of migratory dendritic cells in a rat model of spondyloarthritis. Arthritis Rheum 2012;64:3199–209. 22674414 10.1002/art.34561 PMC3553565

[20] Van der Linden S Valkenburg HA Cats A Evaluation of diagnostic criteria for ankylosing spondylitis. Arthritis Rheum 1984;27:361–8. 6231933 10.1002/art.1780270401

[21] Sallusto F Lenig D Mackay CR Lanzavecchia A Flexible programs of chemokine receptor expression on human polarized T helper 1 and 2 lymphocytes. J Exp Med 1998;187:875–83. 9500790 10.1084/jem.187.6.875 PMC2212187

[22] Annunziato F Cosmi L Santarlasci V Phenotypic and functional features of human Th17 cells. J Exp Med 2007;204:1849–61. 17635957 10.1084/jem.20070663 PMC2118657

[23] Cros J Cagnard N Woollard K Human CD14dim monocytes patrol and sense nucleic acids and viruses via TLR7 and TLR8 receptors. Immunity 2010;33:375–86. 20832340 10.1016/j.immuni.2010.08.012 PMC3063338

[24] Hacquard-Bouder C Falgarone G Bosquet A Defective costimulatory function is a striking feature of antigen-presenting cells in an HLA-B27-transgenic rat model of spondylarthropathy. Arthritis Rheum 2004;50:1624–35. 15146433 10.1002/art.20211

[25] Bowness P Ridley A Shaw J Th17 cells expressing KIR3DL2+ and responsive to HLA-B27 homodimers are increased in ankylosing spondylitis. J Immunol 2011;186:2672–80. 21248258 10.4049/jimmunol.1002653 PMC3210561

[26] Ciccia F Bombardieri M Principato A Overexpression of interleukin-23, but not interleukin-17, as an immunologic signature of subclinical intestinal inflammation in ankylosing spondylitis. Arthritis Rheum 2009;60:955–65. 19333939 10.1002/art.24389

[27] Ciccia F Accardo-Palumbo A Alessandro R Interleukin-22 and IL-22-producing NKp44+ natural killer cells in subclinical gut inflammation of patients with ankylosing spondylitis. Arthritis Rheum 2012;64:1869–78. 22213179 10.1002/art.34355

[28] Appel H Maier R Wu P Analysis of IL-17^+^ cells in facet joints of patients with spondyloarthritis suggests that the innate immune pathway might be of greater relevance than the Th17-mediated adaptive immune response. Arthritis Res Ther 2011;13:R95. 21689402 10.1186/ar3370 PMC3218910

[29] Melis L Van Praet L Pircher H Venken K Elewaut D Senescence marker killer cell lectin-like receptor G1 [KLRG1] contributes to TNF-α production by interaction with its soluble E-cadherin ligand in chronically inflamed joints. Ann Rheum Dis 2014;73:1223–31. 23740233 10.1136/annrheumdis-2013-203881

[30] Melis L Vandooren B Kruithof E Systemic levels of IL-23 are strongly associated with disease activity in rheumatoid arthritis but not spondyloarthritis. Ann Rheum Dis 2010;69:618–23. 19196728 10.1136/ard.2009.107649

[31] Acosta-Rodriguez EV Napolitani G Lanzavecchia A Sallusto F Interleukins 1beta and 6 but not transforming growth factor-beta are essential for the differentiation of interleukin 17-producing human T helper cells. Nat Immunol 2007;8:942–9. 17676045 10.1038/ni1496

[32] McGeachy MJ Chen Y Tato CM The interleukin 23 receptor is essential for the terminal differentiation of interleukin 17-producing effector T helper cells *in vivo*. Nat Immunol 2009;10:314–24. 19182808 10.1038/ni.1698 PMC2945605

[33] Benham H Rehaume LM Hasnain SZ Interleukin-23 mediates the intestinal response to microbial beta-1,3-glucan and the development of spondyloarthritis pathology in SKG mice. Arthritis Rheumatol 2014;66:1755–67. 24664521 10.1002/art.38638

[34] Sherlock JP Joyce-Shaikh B Turner SP IL-23 induces spondyloarthropathy by acting on ROR-γt^+^ CD3^+^CD4^-^CD8^-^ entheseal resident T cells. Nat Med 2012;18:1069–76. 22772566 10.1038/nm.2817

[35] Colbert RA DeLay ML Klenk EI Layh-Schmitt G From HLA-B27 to spondyloarthritis: a journey through the ER. Immunol Rev 2010;233:181–202. 20193000 10.1111/j.0105-2896.2009.00865.x PMC2912611

[36] Turner MJ DeLay ML Bai S Klenk E Colbert RA HLA-B27 up-regulation causes accumulation of misfolded heavy chains and correlates with the magnitude of the unfolded protein response in transgenic rats: implications for the pathogenesis of spondylarthritis-like disease. Arthritis Rheum 2007;56:215–23. 17195225 10.1002/art.22295

[37] Ciccia F Accardo-Palumbo A Rizzo A Evidence that autophagy, but not the unfolded protein response, regulates the expression of IL-23 in the gut of patients with ankylosing spondylitis and subclinical gut inflammation. Ann Rheum Dis 2014;73:1566–74. 23740229 10.1136/annrheumdis-2012-202925 PMC3883901

[38] Campbell EC Fettke F Bhat S Morley KD Powis SJ Expression of MHC class I dimers and ERAP1 in an ankylosing spondylitis patient cohort. Immunology 2011;133:379–85. 21574996 10.1111/j.1365-2567.2011.03453.x PMC3112347

[39] Zeng L Lindstrom MJ Smith JA Ankylosing spondylitis macrophage production of higher levels of interleukin-23 in response to lipopolysaccharide without induction of a significant unfolded protein response. Arthritis Rheum 2011;63:3807–17. 22127699 10.1002/art.30593 PMC3228355

[40] Kenna TJ Lau MC Keith P Disease-associated polymorphisms in ERAP1 do not alter endoplasmic reticulum stress in patients with ankylosing spondylitis. Genes Immun 2015;16:35–42. 25354578 10.1038/gene.2014.62

[41] Neerinckx B Carter S Lories RJ No evidence for a critical role of the unfolded protein response in synovium and blood of patients with ankylosing spondylitis. Ann Rheum Dis 2014;73:629–30. 24106049 10.1136/annrheumdis-2013-204170

[42] Schäkel K Mayer E Federle C A novel dendritic cell population in human blood: one-step immunomagnetic isolation by a specific mAb (M-DC8) and *in vitro* priming of cytotoxic T lymphocytes. Eur J Immunol 1998;28:4084–93. 9862344 10.1002/(SICI)1521-4141(199812)28:12<4084::AID-IMMU4084>3.0.CO;2-4

[43] Schäkel K Kannagi R Kniep B 6-Sulfo LacNAc, a novel carbohydrate modification of PSGL-1, defines an inflammatory type of human dendritic cells. Immunity 2002;17:289–301. 12354382 10.1016/s1074-7613(02)00393-x

[44] Jacques P Venken K Van Beneden K Invariant natural killer T cells are natural regulators of murine spondylarthritis. Arthritis Rheum 2010;62:988–99. 20131252 10.1002/art.27324

[45] Jongbloed SL Lebre MC Fraser AR Enumeration and phenotypical analysis of distinct dendritic cell subsets in psoriatic arthritis and rheumatoid arthritis. Arthritis Res Ther 2006;8:R15. 16507115 10.1186/ar1864 PMC1526567

[46] Hänsel A Günther C Ingwersen J Human slan (6-sulfo LacNAc) dendritic cells are inflammatory dermal dendritic cells in psoriasis and drive strong TH17/TH1 T-cell responses. J Allergy Clin Immunol 2011;127:787–94.e1–9. 21377044 10.1016/j.jaci.2010.12.009

[47] de Baey A Mende I Baretton G A subset of human dendritic cells in the T cell area of mucosa-associated lymphoid tissue with a high potential to produce TNF-alpha. J Immunol 2003;170:5089–94. 12734354 10.4049/jimmunol.170.10.5089

[48] Johansson-Lindbom B Svensson M Wurbel M-A Selective generation of gut tropic T cells in gut-associated lymphoid tissue (GALT): requirement for GALT dendritic cells and adjuvant. J Exp Med 2003;198:963–9. 12963696 10.1084/jem.20031244 PMC2194196

[49] Taurog JD Richardson JA Croft JT The germfree state prevents development of gut and joint inflammatory disease in HLA-B27 transgenic rats. J Exp Med 1994;180:2359–64. 7964509 10.1084/jem.180.6.2359 PMC2191772

[50] Van Praet L Van den Bosch FE Jacques P Microscopic gut inflammation in axial spondyloarthritis: a multiparametric predictive model. Ann Rheum Dis 2013;72:414–7. 23139267 10.1136/annrheumdis-2012-202135

