# Supplementary material for: Ankylosing spondylitis patients display altered dendritic cell and T cell populations that implicate pathogenic roles for the IL-23 cytokine axis and intestinal inflammation
Source: Rheumatology (Oxford). 2015 Aug 28;55(1):120–32. doi: 10.1093/rheumatology/kev245 (PMC4676904; doi:10.1093/rheumatology/kev245)
Supplement: Supplementary Data [file supp_55_1_120__index.html]

Ankylosing spondylitis patients display altered dendritic cell and T cell populations that implicate pathogenic roles for the IL-23 cytokine axis and intestinal inflammation — Ankylosing spondylitis patients display altered dendritic cell and T cell populations that implicate pathogenic roles for the IL-23 cytokine axis and intestinal inflammation — Supplementary Data 

# Ankylosing spondylitis patients display altered dendritic cell and T cell populations that implicate pathogenic roles for the IL-23 cytokine axis and intestinal inflammation

## Supplementary Data

files

- Supplementary Data - docx file
