# Supplementary material for: Ankylosing spondylitis patients display altered dendritic cell and T cell populations that implicate pathogenic roles for the IL-23 cytokine axis and intestinal inflammation
Source: Rheumatology (Oxford). 2015 Aug 28;55(1):120–32. doi: 10.1093/rheumatology/kev245 (PMC4676904; doi:10.1093/rheumatology/kev245)
Supplement: Supplementary Data [file supp_kev245_rhe-14-1650-File008.docx]

**Supplementary Data**

**Supplementary Table S1: Clinical characteristics of AS patient and HC cohorts**

|  | AS Patients | Healthy Controls |
| --- | --- | --- |
| Age, mean ± S.D, yrs  Sex | 54.61 ± 12.05 | 40.83 ± 12.64 |
| Sex, Male/Female, No.  D | 55/12 | 34/15 |
| Disease Duration, mean ± S.D, yrs | 28.17 ± 12.8 | N/A |
| B27, Pos/Neg (% B27^+^) | 64/4 (94%) | 12/36 (24%) |
| BASDAI, mean ± S.D, (score, 0-10) | 3.99 ± 2.24 | N/A |
| BASMI, mean ± S.D, (score, 0-10) | 4.17 ± 2.50 | N/A |
| ESR, mean ± S.D, mm/hr | 12.84 ± 12.18 | N/A |
| CRP, mean ± S.D, mg/L | 7.53 ± 10.29 | N/A |
| Bilateral Sacroiliitis, No. (%) | 53 (76.8%) | N/A |
| Spinal disease |  |  |
| Absent, No. (%) | 17 (24.6%) | N/A |
| Level 1, No. (%) | 12 (17%) | N/A |
| Level 2, No. (%) | 11 (15.9%) | N/A |
| Level 3, No. (%) | 7 (10.1%) | N/A |
| Peripheral Disease |  |  |
| IBD, No. (%)  11.10% | 5 (7.3%) | N/A |
| Uveitis, No. (%) | 4 (5.8%) | N/A |
| Psoriasis, No. (%) | 4 (5.8%) | N/A |
| Arthritis, No. (%) | 11 (15.9%) | N/A |
| Medication |  |  |
| DMARDs, No. (%) | 1 (1.5%) | N/A |
| NSAIDs, No. (%) | 31 (45%) | N/A |
| Combination, No. (%) | 5 (7.3%) | N/A |
| Biologics, No. (%) | 9 (13.0%) | N/A |
| Nil, No. (%) | 6 (8.7%) | N/A |

Percentages for parameters reflect proportion of patient cohort (n=67). Data for the unaccounted proportion was unavailable. Three arthritic AS patients additionally presented with either IBD, psoriasis or uveitis. Combination therapy refers to patients receiving NSAIDs in addition to either DMARD or biological therapy. Spinal disease was categorised according to the presence or absence of cervical, thoracic and lumbar involvement, with levels referring to the number of affected sites: 1 site = level 1, 2 sites = level 2 and 3 sites = level 3. N/A = Not applicable. Mean ± SD is shown. No.: number; HC: healthy controls.

**Supplementary Figure S1: Chemokine profile of circulating CD4+ T cells**


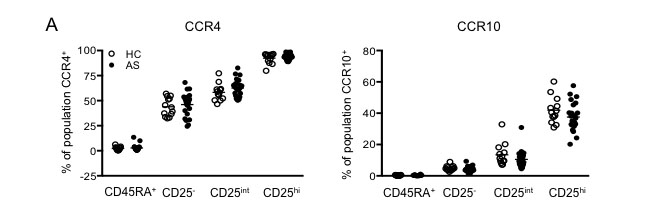


Expression of CCR4 (left) and CCR10 (right) on HC (empty circles, n=9-13) and AS patient (filled circles, n=18-25) CD4+ T cell subsets. CCR+ gates were based on specific isotype. Each dot represents one individual.

**Supplementary Figure S2: Identification of circulating myeloid subsets**


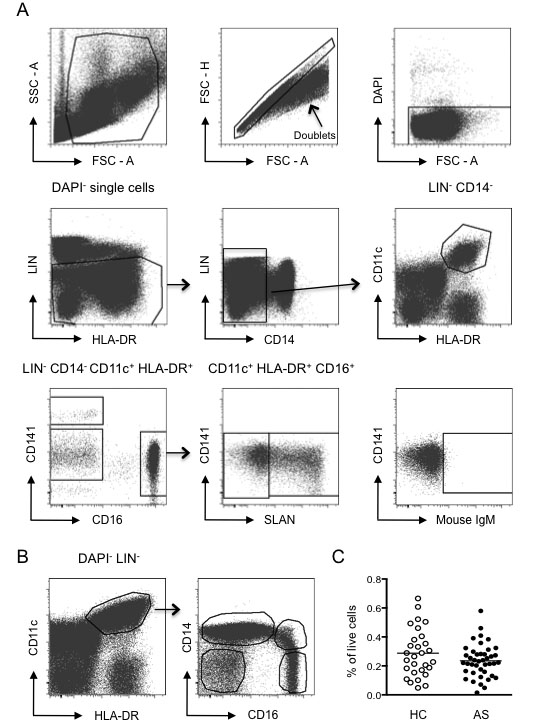


(A) Single live cells (DAPI-) were analysed for expression of LIN markers (CD3/CD15/CD19/CD56). LIN- CD14- CD11c+ HLA-DR+ cells are divided into CD141+ DCs, CD1c+ DCs and CD16+ mononuclear cells. The latter population was heterogeneous for SLAN expression. (B) Depiction of monocytes and DCs within the CD11c+ HLA-DR+ compartment. Live, single cells co-expressing CD11c and HLA-DR were divided into four myeloid populations: CD14+ CD16- monocytes, CD14+ CD16+ monocytes, DCs (CD14- CD16-) and CD14- CD16+ mononuclear cells. (C) Proportion of circulating pDCs (LIN- HLA-DR+ CD123+ CD304+) in HCs (n=29) and AS patients (n=42).

**Supplementary Figure S3: Association between CD141+ DCs and disease severity**


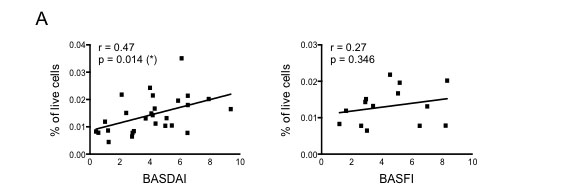


AS patient correlative analyses were performed between frequency of CD141+ DCs and BASDAI (left, n=27) or BASFI (right, n=14) score. Each dot represents one individual. Analyses were performed using linear regression and spearman (r) correlation statistical tests, with the Dunn multiple comparisons post test where * = p <0.05.

**Supplementary Figure S4: HC and AS patient myeloid subset function**


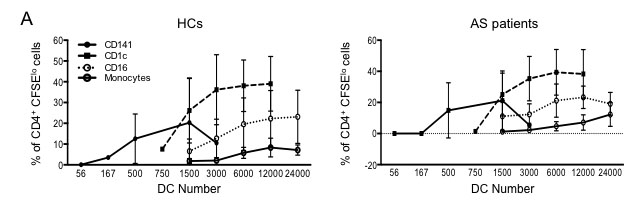


Individual myeloid subsets were cultured with 20,000 naïve CFSE+ CD4+ T cells for 5 days. T cell proliferation was assessed by CFSE dilution. Proportion of proliferating T cells induced following co-culture with HC and AS patient CD141+ and CD1c+ DCs, CD14- CD16+ mononuclear cells and CD14+ monocytes was compared. Error bars represent mean ± SD. Numbers used for analysis: - CD141+ DCs (8 HCs/13 AS patients), CD1c+ DCs (10 HCs/16 AS patients), CD14- CD16+ mononuclear cells (9 HC/15 AS patients) and CD14+ monocytes (4 HC/6 AS patients). HC: healthy controls.
